# The Screening and Mechanism of Influenza-Virus Sensitive MDCK Cell Lines for Influenza Vaccine Production

**DOI:** 10.3390/diseases12010020

**Published:** 2024-01-10

**Authors:** Zhaona Yang, Shouzhi Yu, Ying Xu, Yuxiu Zhao, Lili Li, Jingjie Sun, Xin Wang, Yancen Guo, Yuntao Zhang

**Affiliations:** Beijing Institute of Biological Products Company Limited, Beijing 100176, China; yangzhaona2020@163.com (Z.Y.); xuying_beans@163.com (Y.X.); zhao10306@126.com (Y.Z.); lilili43@sinopharm.com (L.L.); 13126588030@163.com (J.S.); wss12jj@163.com (X.W.); nancyguo1112@sina.com (Y.G.)

**Keywords:** MDCK, influenza virus, influenza vaccine, H1N1, H3N2, BV, BY

## Abstract

Influenza is a potentially fatal acute respiratory viral disease caused by the influenza virus. Influenza viruses vary in antigenicity and spread rapidly, resulting in seasonal epidemics. Vaccination is the most effective strategy for lowering the incidence and fatality rates of influenza-related disorders, and it is also an important method for reducing seasonal influenza infections. Mammalian Madin–Darby canine kidney (MDCK) cell lines are recommended for influenza virus growth, and such cell lines have been utilized in several commercial influenza vaccine productions. The limit dilution approach was used to screen ATCC-MDCK cell line subcellular strains that are especially sensitive to H1N1, H3N2, BV, and BY influenza viruses to increase virus production, and research on influenza virus culture media was performed to support influenza virus vaccine development. We also used RNA sequencing to identify differentially expressed genes and a GSEA analysis to determine the biological mechanisms underlying the various levels of susceptibility of cells to influenza viruses. MDCK cell subline 2B6 can be cultured to increase titer and the production of the H1N1, H3N2, BV, and BY influenza viruses. MDCK-2B6 has a significantly enriched and activated in ECM receptor interaction, JAK-STAT signaling, and cytokine receptor interaction signaling pathways, which may result in increased cellular susceptibility and cell proliferation activity to influenza viruses, promote viral adsorption and replication, and elevate viral production, ultimately. The study revealed that MDCK-2B6 can increase the influenza virus titer and yield in vaccine production by increasing cell sensitivity and enhancing proliferative activity.

## 1. Introduction

Influenza is a potentially fatal acute respiratory viral disease caused by the influenza virus. Influenza viruses vary their antigenicity and spread quickly, creating seasonal epidemics and even pandemic outbreaks, resulting in 3–5 million severe cases, 300,000 to 600,000 fatalities, and considerable economic losses annually [1]. The currently recognized best method for preventing influenza is vaccination. According to the final immunization plan set by the Global Influenza Vaccine Action Plan, the global annual demand for influenza vaccines is approximately 10.78 billion doses (calculated based on 70% of the world population and two doses of immunization) [2]. According to WHO data, the global production capacity of seasonal influenza vaccines and pandemic influenza vaccines in 2019 was 1.48 billion doses and 8.31 billion doses, respectively, accounting for only 13.7% and 77.1% of the demand [3,4]. The current production capacity of influenza vaccines is insufficient, and there is an urgent need to develop novel and efficient production methods for influenza vaccines. Compared to traditional chicken embryo production processes, large-scale animal cell culture technology to produce influenza vaccines has become an essential focus of influenza vaccine process development in recent years [5]. Utilizing the critical technology of a large-scale animal cell culture to increase the production of influenza virus is the key to establishing an efficient and economical influenza virus production process.

Currently, seasonal influenza vaccines for human use are mostly trivalent or tetravalent, including subtypes H1N1 and H3N2, as well as one type of influenza B virus (trivalent) or two types of influenza B virus (tetravalent) [3]. The seasonal influenza virus strains are recommended annually by the WHO for both the northern and southern hemispheres, and the isolated strains are distributed to manufacturers for production [3,4]. Currently, human influenza vaccines that have obtained production licenses and marketed in different countries or regions include whole virus or lytic inactivated vaccines, such as Vaxigrip from Sanofi Pasteur [6], Flucalvax from Seqirus Australia [7], SKYCellflus from SK Chemicals in South Korea [8], attenuated live vaccine of FluMist from Medlmmune [9], and the recombinant protein vaccine of Flublok from Sanofi Pasteur [10]. Among all the vaccines, inactivated vaccines are the most popular products. In addition, many new technologies to develop influenza vaccines such as VLP (virus-like particles), virus vectors, and nucleic acid vaccines have grown in recent years, and some achievements have entered the clinical trial stage.

Influenza vaccines are still mainly produced using chicken embryo technology, which has a history of over 70 years. Although this technology has been widely adopted for automated production, the improvement of overall production capacity and the uniformity of production and quality worldwide are still severely limited due to its many drawbacks, such as the need for a sufficient supply of chicken embryos; the significant individual differences in chicken embryos being a result of batch differences in virus production and immune efficacy; chicken embryo raw materials being prone to the presence of exogenous pathogens; some people being allergic to chicken embryo protein; and the fact that they are easily mismatched with antigens of widespread isolates. To overcome the shortcomings of chicken embryo technology, animal cells have become a new focus of attention as a production substrate [11]. Compared with chicken embryo technology, the influenza virus produced based on animal cell culture technology grows better and has a higher antigen matching degree with the original virus strain, overcoming the problem of people being allergic to egg ingredients. The process is controllable and easy to standardize or scale up, which can not only improve the production efficiency and effectiveness of influenza vaccines, but also be more conducive to potential influenza pandemic outbreaks and quickly produce pandemic influenza vaccines in a short period. Therefore, organizations such as the WHO and governments from multiple countries encourage animal cell culture technology to replace traditional chicken embryo technology in producing influenza vaccines [12].

Currently, chicken embryo vaccines account for 85% to 90% of the inactivated influenza vaccines on the market share. In comparison, animal cell culture products only account for 10% to 15%, indicating the vast market development potential of the latter. At the moment, immortal or modified cell lines capable of continuous cultivation, such as the Madin–Darby Canine Kidney (MDCK), the African green monkey renal epithelial cell line (Vero), HEK293, and others, are employed in the manufacturing of influenza vaccines [13]. In 1995, the World Health Organization (WHO) advised that mammalian cell lines should develop the influenza virus culture, including the MDCK, Vero, and the human retinal-derived cell line (PER.C6 cells) [14]. Since then, cell culture influenza vaccine research has exploded, among which MDCK cells have become the primary substrate for cell-based influenza vaccines, due to the low amplification variance, high viral titer, and efficient amplification [15,16,17,18].

For producers to develop and supply influenza vaccines, the WHO formally issued the recommended components for the 2023–2024 northern hemisphere seasonal influenza vaccine on 24 February 2023. The following are the influenza virus strains for which cell culture or recombinant-based vaccinations are advised in the northern hemisphere: a B/Austria/1359417/2021 (B/Victoria lineage)-like virus (BV), a B/Phuket/3073/2013 (B/Yamagata lineage)-like virus (BY), an A/Wisconsin/67/2022 pdm09-like virus (H1N1), an A/Darwin/6/2021-like virus (H3N2) [19,20].

In China, a significant area of vaccine research and development is the production of the influenza vaccine by the MDCK cell matrix. However, because of the MDCK’s notable changes in cell shape, proliferation rate, and virus sensitivity, cell safety restricted its application in large-scale vaccine production. Currently, most MDCK cells have generated from ATCC and many laboratories have used subcellular strains to meet various needs. Companies of Novartis and MeddImmune have obtained MDCK cell lines with their independent intellectual property rights and are qualified to produce influenza vaccines through cell subcloning and screening [21].

The selection of cell lines to manufacture influenza vaccines can affect many process aspects such as virus yield, bioreactor configuration, and purification efficiency. High-yield MDCK cell lines can be screened and generated by combining cell screening approaches with developing technologies, such as metabolomics, RNA sequencing analysis, and gene-editing. This study selected MDCK cell sublines that are sensitive to four types of influenza virus (H1N1, H3N2, BV, and BY), provide vital cell matrix research for the manufacturing of influenza vaccines, and reveal the influence of culture medium components on the stability of virus passage. Meanwhile, this study investigates the molecular mechanisms underlying the varying sensitivities of distinct MDCK cell clones to influenza viruses by RNA sequencing, providing a direction for fundamental research and influenza vaccine development.

## 2. Materials and Methods

### 2.1. Cells

ATCC-MDCK cells were derived from the United States ATCC and frozen by the Beijing Institute of Biological Products Co., Ltd. (Beijing, China).

### 2.2. Influenza Viruses

The influenza strains of a B/Austria/1359417/2021 (B/Victoria lineage)-like virus (BV), a B/Phuket/3073/2013 (B/Yamagata lineage)-like virus (BY), an A/Wisconsin/67/2022 pdm09-like virus (H1N1), and an A/Darwin/6/2021-like virus (H3N2) that are recommended for vaccine use by the WHO were stored in the Beijing Institute of Biological Products Co., Ltd. (Beijing, China).

### 2.3. Construction of MDCK Cell Subclones by the Limit Dilution Method

We renewed the MDCK cells, digested the cells according to the cell passage steps when the cell convergence was about 80%, blew and fully dispersed the cells to form a single cell suspension, sampled and counted them, and diluted the cells to a final concentration of 10 cells/mL. We entirely mixed the suspension and inoculated the cells into a 96-well plate, with 50 μL per well. We placed a 96-well plate in a 5% CO_2_ incubator at 37 °C for 24 h and observed whether each well was a single cell. After 3 days of incubation, we added 50 μL of preheated cell growth fluid to each well. After 7 days, we observed it under a microscope to determine whether cell clumps were composed of cells of uniform shape and size. When the cells covered 50% of the bottom area of the well, we conducted cell passage and expansion.

### 2.4. Hemagglutination Titer Test

We added 10 μL of the influenza virus culture supernatant to the V-type hemagglutination plate, added an appropriate amount of PBS, and successively conducted a twofold gradient dilution to produce a final volume of 25 μL. We added an equal volume of 1% chicken red blood cells to each well, shook the solution well, and let it stand at room temperature for 45 min. The observation result “−” indicates no agglutination with the deposit of red blood cells at the bottom of the tube, forming a circular disk with neat edges. “+” indicates microagglutination with red blood cells deposited at the bottom of the tube, forming a circular disk with unclear edges. “++” indicates agglutination with red blood cells deposited in a circular shape at the bottom of the tube, surrounded by agglutinated small pieces. “+++” indicates primarily agglutinates, with red blood cells presenting granular agglutination and a drooping trend at the edges. “++++” indicates complete agglutination with red blood cells lying flat on the bottom of the tube in a reticular pattern. To determine the titer, the control tube should not agglutinate, and the experimental tube should be observed to classify the maximum dilution of the virus that can cause “++” agglutination as the hemagglutination titer. This experiment was repeated 3 times for all the samples.

### 2.5. Screening of Influenza-Virus-Sensitive MDCK Subclonal Cells

When the subclone MDCK cell’s confluence reached 70% to 90%, we discarded the culture medium and washed it twice with PBS to properly inoculate it with the influenza virus. We added 0.5 μg/mL of TPCK trypsin and incubated it in a 37 °C, 5% CO_2_ incubator. We observed the pathological changes in the cells every day and took samples for hemagglutination titer testing after 96 h. We took the top five subcellular clones with the highest hemagglutination titers from each batch of cells and froze these cells for storage.

### 2.6. Cell Proliferation Ability Detection by CCK-8

Inoculate cell suspension (100 μL/well) in a 96-well plate. Pre-incubate the plate at 37 °C, 5% CO_2_. Add 10 µL of the Cell Counting Kit-8 solution to each well of the plate. Incubate the plate for 2 h in the incubator. Measure the absorbance at 450 nm using a microplate reader. Data are means ± SD. Two-tailed Student’s *t*-test determined statistical significance.

### 2.7. Cell Proliferation Activity Detection by Flow Cytometry

Cells were cultured at 37 °C, 5% CO_2_ for 120 h. To evaluate MDCK cell proliferation activity, cell surface staining FITC anti-ki67 antibody (eBioscience, San Diego, CA, USA, 11-5699-42, 5 µL/test) was performed at room temperature in the dark for 30 min, and ki67 positive MDCK cells were sorted by flow cytometry. FCS EXPRESS (FCS Express™, Pasadena, CA, USA) or FlowJo 10.8.1 (FlowJo, Ashland, OR, USA) Software and was used for data analysis.

### 2.8. Gene Set Enrichment Analysis (GSEA)

RNA samples were extracted from MDCK-2B6 and MDCK-Ctrl cells using the RNeasy^®^ Mini Kit (Qiagen, Hilden, Germany) and sequenced using an Illumina HiSeq2500 sequencer (Illumina, San Diego, CA, USA) to obtain 100 bp paired-end reads. The gene expression level was measured with Cufflinks v2.1.1. The noncoding region was removed. Multiread correction and frag-bias-correct were used to improve the measurement accuracy. Differentially expressed genes (DEGs) were identified using the Cuffdiff tool with a statistically significant q-value (<0.05). A Gene Set Enrichment Analysis (GSEA) was performed to analyze the critical transcriptome pathways, and the estimated expression levels were used in the GSEA to determine the enrichment scores according to the ranked-order gene list. The pathways containing at least 15 genes were evaluated. The significant scores were computed using the 1000 nonparametric permutation test, and *p* values < 0.05 were considered statistically significant.

## 3. Results

### 3.1. The First-Round Screening of Influenza-Virus-Sensitive MDCK Subclonal Cells

**Figure 1:**
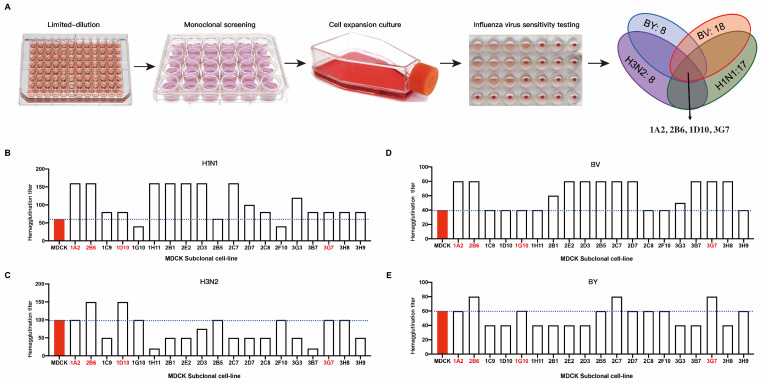
Sensitivity of MDCK subclone cells to the influenza virus. (**A**) Schematic diagram of the subclone isolation and screening process for MDCK cells; MDCK cell clones with increased sensitivity to four influenza viruses underwent subsequent screening. Hemagglutination titers of different subclones to influenza viruses of H1N1 (**B**), H3N2 (**C**), BY (**D**), and BY (**E**).

We subcloned recently revived P59 generation ATCC-MDCK cells using the limit dilution technique. Four influenza viruses, H1N1, H3N2, BV, and BY, were each introduced into the subcloned cells. We measured the hemagglutination titer when 75% of the cells were cytopathic. The outcomes demonstrated that the susceptibility of various MDCK cell clones to various influenza viruses varied (Figure 1A). We chose clones with high hemagglutination titers to target H1N1. These were 1A2, 2B6, 1C9, 1D10, 1H11, 2B1, 2E2, 2D3, 2B5, 2C7, 2D7, 2C8, 3G3, 3B7, 3G7, 3H8, and 3H9 (Figure 1B). The H3N2 clones included 1A2, 2B6, 1D10, 1G10, 2B5, 2F10, 3G7, and 3G8 (Figure 1C). The BV-targeting clones included 1A2, 2B6, 2B1, 2E2, 2D3, 2B5, 2C7, 2D7, 3G3, 3B7, 3G7, and 3H8 (Figure 1D). The clones that targeted BY were 1A2, 2B6, 2C7, 2D7, 2C8, 2F10, 3G7, and 3H9 (Figure 1E). Because the purpose of this study was to screen cell clones that are susceptible to all four influenza viruses (H1N1, H3N2, BV, and BY) at the same time, a cross-analysis of the above clones was performed. The clones selected after the first round of screening were 1A2, 2B6, 2B5, and 3G7.

### 3.2. The Re-Screening of ATCC-MDCK Subclone Cells

**Figure 2:**
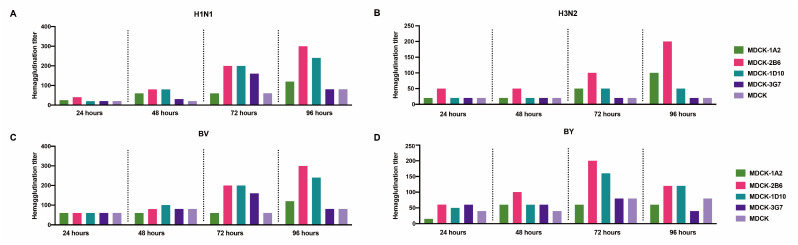
Second screening of candidate subclone cells. (**A**) Hemagglutination titers of the H1N1, (**B**) H3N2, (**C**) BV, and (**D**) BY influenza viruses at different infection times in MDCK and MDCK subclones.

We carried out a second-round screening comparison on ATCC-MDCK and its 1A2, 2B6, 2B5, and 3G7 subclones to choose favorable MDCK cell clones further. After the virus was inoculated, the culture supernatant’s hemagglutination titers were assessed at 24, 48, 72, and 96 h. The analysis of the five cell lines’ sensitivity to the four distinct influenza viruses revealed that the titer of the virus continuously increased during cultivation, reaching its peak at 72 or 96 h. Compared with MDCK and the 1A2, 2B5, and 3G7 subclones, MDCK-2B6 had the highest level of sensitivity. Following the infection of MDCK-2B6, the titers of the H1N1, H3N2, and BV viruses peaked at 96 h, while those of the BY virus peaked at 72 h (Figure 2). These data suggest that we should adjust the harvesting time of the virus stock according to different virus types during process scaling up during production.

### 3.3. A Comparison of Influenza Virus Culture Media

**Figure 3:**
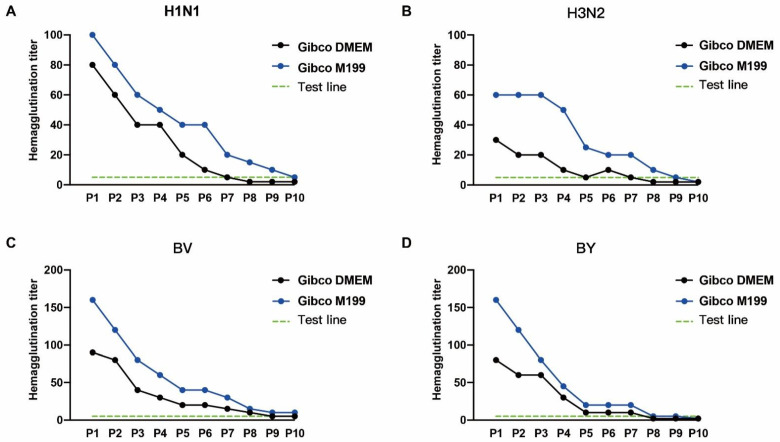
The impacts of cell culture media on the influenza virus passage. The hemagglutination titers of the (**A**) H1N1, (**B**) H3N2, (**C**) BV, and (**D**) BY influenza viruses with different passage numbers of MDCK-2B6 cells.

A critical element that influences viral development is the culture media. We compared the impacts of Gibco DMEM (CAT# 12800-017) and Gibco M199 (CAT# 31100-035) on the titers of four viruses in MDCK-2B6 cells to establish the best growth medium for this purpose. The influenza virus hemagglutination titer discovered at 72 h after the virus injection in MDCK-2B6 cells. The findings revealed that the H1N1, BV, and BY titers were more significant in the M199 maintenance solution, whereas there were no discernible differences in the H3N2 titers (Figure 3). To assess the stability of the virus passage, the virus was constantly passed under two distinct media conditions. The results showed that the stability of the influenza virus reduced as the passage number of subcultures increased in both media, but the virus titer remained higher in the M199 medium than in DMEM, indicating that the M199 medium is better suited for influenza virus growth.

### 3.4. The Mechanism of MDCK-2B6 Sensitivity to Influenza Viruses

**Figure 4:**
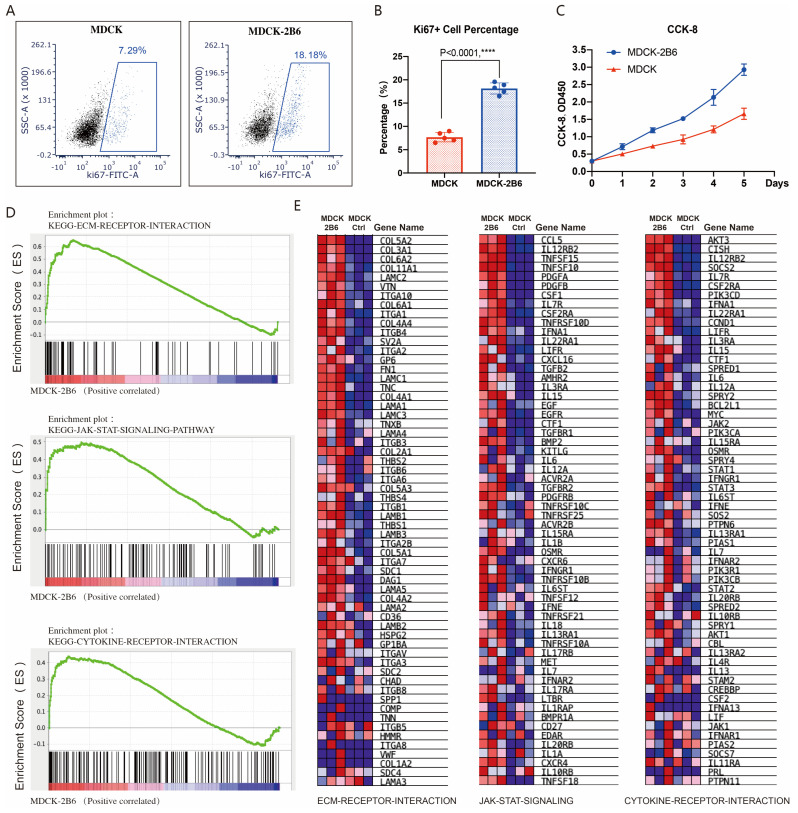
Cell proliferation activity affects viral titer. (**A**) The proportion of Ki67-positive cells detected by flow cytometry. (**B**) Differences in the proportion of Ki67-positive cells in statistics. (**C**) CCK-8 method for detecting cell proliferation ability. (**D**) RNA-Seq and signaling pathway enrichment analysis. Molecular signature classification screening by GSEA analysis with top enriched with representative ECM receptor interaction signaling, JAK-STAT signaling, and cytokine receptor interaction signaling pathways. (**E**) Differential expression gene heatmap in the ECM receptor interaction signaling, JAK-STAT signaling, and cytokine receptor interaction signaling pathways. Data are means ± SD. Two-tailed Student’s *t*-test determined statistical significance. *p* < 0.0001, ****.

Two key factors affect virus production: cell sensitivity to viruses and cell proliferation ability. We also tested the difference in proliferation ability between MDCK cells and MDCK-2B6 cells. Using flow cytometry to detect the level of KI67, a marker protein for cell proliferation activity after culture, it was found that the proportion of KI67-positive cells in MDCK-2B6 cells was significantly higher than that in 2B6 cells (Figure 4A,B). By using the CCK-8 method to detect the proliferation curve of cells, it was found that MDCK-2B6 cells have a faster proliferation rate, resulting in a higher cell density than MDCK (Figure 4C). Research has shown that influenza virus infections of cells can cause changes in host cell signaling pathways, such as mitogen-activated protein kinase (MAPK), phosphatidylinositol-3-kinase/protein kinase B (PI3K/Akt), the nuclear factor kappa light chain enhancer of activated B cells (NF–κB), and Toll-like receptors/RIG-I-like receptors (TLR/RIG), and other factors can also cause changes in the expression level of cytokines. To investigate why the titer of the influenza virus in MDCK-2B6 cells is higher than that in MDCK cells, we extracted RNA samples from two cell lines and performed RNA seq detection. We used the GSEA to perform a signal pathway enrichment analysis on differentially expressed genes. The results showed that, in MDCK-2B6 cells, the extracellular matrix (ECM) receptor interaction, JAK-STAT signaling, and cytokine receptor interaction signaling pathways (Figure 4D,E) were significantly enriched and activated. Interactions of the ECM and cellular receptors constitute a crucial pathways involved in cell growth and metabolism. The ECM–cell interactions are mediated directly or indirectly via cell surface receptors with cooperative molecules. The Janus kinase/signal transducers and activators of transcription (JAK/STAT) signaling pathway is a universally expressed intracellular signal transduction pathway involved in many crucial biological processes, including cell proliferation, differentiation, apoptosis, and immune regulation. It provides a direct mechanism for extracellular-factor-regulated gene expression. Cytokines are a broad category of intercellular signaling proteins that act in almost every aspect of cell activity. Changes in these pathways may lead to the increased sensitivity of cells to influenza viruses, easier virus adsorption and replication, and, ultimately, increased virus production. The differentially expressed genes in the above signaling pathways are shown in Figure 4D,E.

## 4. Discussion

One of the key processes for inactivating influenza vaccines is the viral titer during the cultivation process, which significantly affects the sensitivity and density of cells. This study used the limit dilution method to screen MDCK cell clones sensitive to all four widely prevalent influenza viruses; candidate cells MDCK-2B6 were obtained through secondary screening. By detecting the effects of two culture media on virus titers, it was determined that M199 is more suitable for amplifying influenza viruses in 2B6 cells. However, virus titers still show a downward trend with passage times. In addition to the sensitivity of MDCK cells leading to changes in the virus titer, this study also detected changes in the proliferation ability of MDCK cells. It was found that 2B6 cells increased their proliferation rate and cell density through changes in multiple signaling pathways, further increasing the production of the influenza virus. The above research provides a cell matrix screening method for influenza vaccine production.

The manufacturing of influenza vaccines utilizing animal cell culture technology has demonstrated considerable benefits and has been identified as the primary research and development focus for the next generation of influenza vaccinations [22]. However, this technology still needs to be fully mature, and several cultivation methods have flaws that prevent their widespread use. There is still room for improvement and advancement. Differences in cell lines and culture circumstances, particularly in the transition from serum-containing medium to serum-free medium, may have a major impact on the cell properties [23,24].

The physiological activities of cell populations may be compromised during long-term, large-scale artificial cultivation, such as diminished or lost secretory and metabolic capacities. It is currently unknown whether certain animal cell lines may amplify various subtypes of influenza viruses without changing their genetic or antigenic properties [25]. The identification and testing of cell libraries, and the development of cell substrates suited for the replication and proliferation of diverse vaccine virus strains, are required. Furthermore, animal cells are vulnerable to pathogen infection from respiratory viruses, enteroviruses, and herpesviruses, necessitating stricter quality control to ensure the cell-matrix vaccination’s safety. The future development of animal cell culture technology should focus on optimizing the cell culture environment and improving cell features to enhance production scale and vaccine manufacturing [26]. Adopting a specific vaccine production system, on the other hand, may not be sufficient, especially in a pandemic setting, where the demand may be impossible to fulfill. In addition to traditional vaccinations based on chicken embryos and cell-matrix cultures, there will always be some reliance on technological platforms regarding the antigenic structure design, nanoparticles, and viral vectors [27].

Some influenza viruses have a low replication efficiency in MDCK cells. Progress has also been made in the genetic alteration of MDCK cells to increase the sensitivity of influenza viruses. Cells can be changed to boost their output in addition to creating high-yield and stable vaccination strains by classical recombination or reverse genetics. The MDCK-SIAT1 cell line is more suitable for the isolation or amplification of clinical influenza samples if it is passed through human-stable transfection of 2,6-neneneba sialic acid transferred cDNA into the MDCK cells-2,6-Connect Sialic acid receptor; however, the MDCK London cell line, constructed by the Common Cold Laboratory in Salisbury, England, grows faster and is sensitive to a variety of influenza virus strains [28,29]. In the absence of toluene phenylalanine chloromethyl ketone (TPCK) trypsin (trypsin), it enables the multicycle development of the H5 and H9 subtypes of the avian influenza virus, and the virus titer is equivalent to that of the virus growing in MDCK cells with added TPCK trypsin [30]. Guo created a stable MDCK cell line with tissue transglutaminase 2 (TGM2) overexpression and knockdown and investigated the effect of TGM2 on influenza A (H1N1) virus growth in MDCK cells. The results showed that knocking down the TGM2 gene boosted the expression of the viral nucleoprotein (NP) gene and raised the virus titer considerably [31].

Vaccination is now the most effective approach to preventing influenza, which remains a severe public health danger. However, the influenza virus epidemic scenario varies annually due to the rapid respiratory transmission and high frequency changes. To effectively control seasonal influenza and any future pandemics, it is critical to research and develop a universal influenza vaccine that can respond to all types and subtypes of influenza viruses and induce long-lasting immunity. [32]. Although we have completed efficient MDCK cell clone screening, further research on the tumorigenicity and exogenous factors of the subclone strain MDCK-2B6 is needed. Whether MDCK-2B6 can used in industrial production still requires comprehensive testing. The research on virus cultivation technology is limited to small-scale experiments, and the selection of a culture medium of actual production still requires reactor-scale validation. In summary, this study focused on cell line optimization and culture medium optimization, providing important experimental support for the rapid production of influenza vaccines.

## Data Availability

The data presented in this study are available within the article.
